# Cutaneous Sinus Formation Is a Rare Complication of Thyroid Fine Needle Aspiration Biopsy

**DOI:** 10.1155/2014/923438

**Published:** 2014-12-08

**Authors:** Gülhan Akbaba, Muhyettin Omar, Murat Polat, Önder Özcan, Ahmet Korkut Bellı, Murat Şahan, Neşat Çullu

**Affiliations:** ^1^Department of Endocrinology and Metabolism Diseases, Faculty of Medicine, Muğla Sıtkı Koçman University, 48000 Mugla, Turkey; ^2^Department of Internal Medicine, Faculty of Medicine, Muğla Sıtkı Koçman University, 48000 Mugla, Turkey; ^3^Department of General Surgery, Faculty of Medicine, Muğla Sıtkı Koçman University, 48000 Mugla, Turkey; ^4^Department of Ear, Nose and Throat Diseases, Faculty of Medicine, Muğla Sıtkı Koçman University, 48000 Mugla, Turkey; ^5^Department of Radiodiagnostics, Faculty of Medicine, Muğla Sıtkı Koçman University, 48000 Mugla, Turkey

## Abstract

Fine needle aspiration biopsy (FNAB) is essential in the diagnosis and management of thyroid nodules. In this paper, we report a rare complication, cutaneous sinus formation, after diagnostic FNAB guided by palpation. Sixty-three-year-old female patient was admitted with the complaints of hoarseness and discharge from the anterior neck wall which were present for the last 6 months. The patient underwent a near total thyroidectomy 17 years ago. Recurrent nodular goiter was detected six months before and a diagnostic FNAB guided by palpation was performed. Two weeks later the patient had wound discharge and hoarseness. Physical examination of the patient revealed a sinus, which was located superior to the thyroidectomy incision. A 1 cm nodule was palpated in the left side of her neck. A cervical ultrasonography (USG) showed a 9 × 7 mm nodule in the remnant thyroid and a 9.5 × 3.5 mm fistulized fluid collection. The patient underwent sinus tract and remnant thyroid removal. This case report presents a cutaneous sinus formation deriving from the granulation tissue, probably due to the silk suture reaction in the previous surgery, by the FNAB guided by palpation procedure. We suggest USG guided FNAB to achieve more accurate and safe diagnosis in evaluating the thyroid nodules.

## 1. Introduction 

Multinodular goitre (MNG) is one of the most common diseases of endocrine disorders. The incidence of palpable thyroid nodules is 3–7% and more than 50% of the population have thyroid nodules detected with ultrasonographic (USG) examination [1]. Noninvasive methods such as radionuclide thyroid scan and thyroid USG have been used for the diagnosis of thyroid nodules for decades. Fine needle aspiration biopsy (FNAB) guided by USG or palpation is the most feasible diagnostic tool for the thyroid nodule evaluation due to its simplicity, accuracy, and cost-effectiveness [2, 3]. However, like other invasive procedures, FNAB may cause various complications. In this paper, we report a rare complication, cutaneous sinus formation, after diagnostic FNAB guided by palpation.

## 2. Case

**Figure 1:**
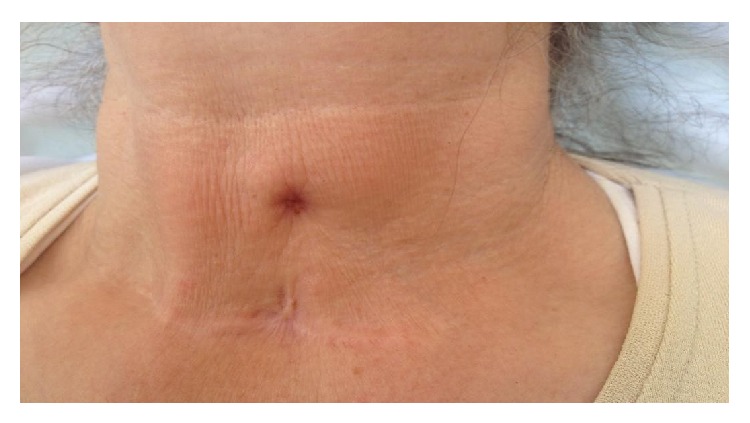
Cutaneous sinus located superior to the thyroidectomy incision.

**Figure 2:**
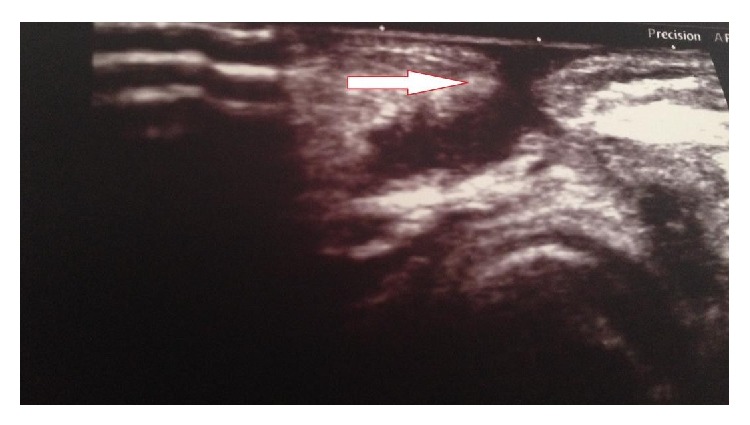
Ultrasonographic image of the subcutaneous sinus tract.

Sixty-three-year-old female patient was admitted to the Department of Endocrinology of Muğla Sıtkı Koçman University Research Hospital with the complaints of hoarseness and discharge from the anterior neck wall which were present for the last 6 months. The patient underwent a near total thyroidectomy 17 years ago but no surgery or pathology records were available. The neck ultrasound showed recurrent nodular goiter and a diagnostic FNAB guided by palpation was performed six months before. Two weeks later the patient had wound discharge and hoarseness which was not improved by a course of antibiotic therapy and was referred to our hospital. Physical examination of the patient revealed a sinus opening with seropurulent discharge located superior to the thyroidectomy incision and the tissue surrounding the sinus opening was moderately swollen and was hyperemic (Figure 1). A 1 cm nodule was palpated in the left side of her neck. No cervical lymph adenopathy or any other systemic finding was found. White blood cell count, neutrophil, eosinophil, C reactive protein, sedimentation rate, liver and thyroid function tests, and antithyroid antibodies were all in normal range. Culture of the discharge did not grow any bacteria. A cervical USG showed a 9 × 7 mm nodule with mixed echogenicities in the left remnant thyroid tissue and a 9.5 × 3.5 mm fistulized fluid collection (Figure 2).

Cervical MRI revealed a 10 × 6 mm subcutaneous tract that was opening to the skin and a minimal fluid intensity that was consistent with postoperative granulation tissue. The patient underwent surgery and the exploration revealed that the sinus tract was ending near the berry ligament close to the left thyroid cartilage and left recurrent laryngeal nerve with silk suture remains. The left recurrent laryngeal nerve was intact; however, it was thickened comparing to the right. The sinus tract and the remnant thyroid tissue were removed with laryngeal nerve monitoring. The pathology report showed that the sinus was associated with chronic inflammation, granulation tissues and adenomatous hyperplasia was present in the remnant thyroid.

## 3. Discussion

Fine needle aspiration biopsy is essential in the diagnosis and management of thyroid nodules. In practice, 25 Gauge needles are recommended to obtain sufficient tissue sample during thyroid needle biopsy. Since FNAB can distinguish the benign nodules from malignant nodules, the need for surgical removal of benign thyroid nodules has been decreased and surgery has become the choice for more selective patients [2, 3]. Traditionally, FNAB has been used to obtain cells for cytologic diagnosis, supplemented by immunocytochemistry. More recently, as noted in later articles in this issue, FNAB has been used to obtain material for genetic and molecular testing [4–6].

Local pain and minor hematomas are the most common clinical complications. Most hematomas are self-limiting and do not cause serious problems [7, 8]. Post-FNAB hemorrhage and fibrosis are the most common histological alterations observed in surgical specimens [9]. In the literature, there are a few reports regarding subcutaneous nodule formation due to malignant cell implantation after FNAB, and this was accepted as a rare cause of thyroid cancer recurrence [10]. Besides, other rare complications have also been reported as case reports. These are intrathyroidal massive hemorrhage and upper respiratory tract congestion [11], nodule infarction [12], recurrent laryngeal nerve palsy [13], tracheal injury related hemoptysis, thyrocutaneous sinus formation [14], and pleural injury resulting in pneumothorax [15]. Recent studies showed that using USG guided FNAB has significantly decreased the ratio of insufficient cytology reports comparing to the palpation guided FNAB [16–18]. Although palpation guided FNAB continues to be a successful approach to the evaluation of palpable lesions, ultrasound guided FNAB enhances precision, documentation, and diagnostic yield in nonpalpable and even in palpable masses.

This case report presents a cutaneous sinus formation deriving from the granulation tissue, probably due to the silk suture reaction in the previous surgery, by the FNAB procedure. We do not know the properties of the FNAB needle, using thick needle to perform FNAB may lead to subcutaneous can fistulas. Since the physician can easily see the target nodule and the surrounding tissue in the USG guided FNAB, it presents a safe diagnostic procedure and decreases the complication ratios. Moreover, USG devices can be found in the majority of the hospitals. Therefore, we suggest USG guided FNAB to achieve more accurate and safe diagnosis in evaluating the thyroid nodules.
